# Bioengineering the ovary to preserve and reestablish female fertility

**DOI:** 10.21451/1984-3143-AR2018-0099

**Published:** 2020-05-22

**Authors:** Fulvio Gandolfi, Matteo Ghiringhelli, Tiziana A.L. Brevini

**Affiliations:** 1 Department of Agricultural and Environmental Sciences - Production, Landscape, Agroenergy, Università degli Studi di Milano, Milano 20122, Italy.; 2 Department of Health, Animal Science and Food Safety, Università degli Studi di Milano, Milano 20122, Italy.

**Keywords:** cryopreservation, decellularization, female infertility, ovary, tissue engineering, 3D printing

## Abstract

Different bioengineering strategies can be presently adopted and have been shown to have great potential in the treatment of female infertility and ovarian dysfunction deriving from chemotherapy, congenital malformations, massive adhesions as well as aging and lifestyle. One option is transplantation of fresh or cryopreserved organs/fragments into the patient. A further possibility uses tissue engineering approaches that involve a combination of cells, biomaterials and factors that stimulate local ability to regenerate/ repair the reproductive organ. Organ transplant has shown promising results in large animal models. However, the source of the organ needs to be identified and the immunogenic effects of allografts remain still to be solved before the technology may enter the clinical practice. Decellularization/ repopulation of ovary with autologous cells or follicles could represent an interesting, still very experimental alternative. Here we summarize the recent advancements in the bioengineering strategies applied to the ovary, we present the principles for these systems and discuss the advantages of these emerging opportunities to preserve or improve female fertility.

## Introduction

Ovary dysfunction and failure is one of the main causes for infertility, with an alarming incidence of one out of 1000 women, under the age of 30, rising to one% in women before 40 (Hewlett and Mahalingaiah, 2015; Qin *et al*., 2015). Patients affected are not able to undergo physiological cycles and/or release oocytes nor they produce normal levels of hormones. Several potential causes have been described. Radio-therapy and/or chemotherapy, viral infections, environmental factors, metabolic and autoimmune diseases, and genetic alterations (Yalcinkaya *et al*., 2014; Sadri-Ardekani and Atala, 2015; Qin *et al*., 2015). Studies performed in the last years have shown for instance that the use of alkylating agents associated with abdominal ionizing radiation will render infertile almost 100% of the patients prepared for bone marrow transplantation (Wallace *et al.*, 2005; Anderson and Wallace, 2011; Donnez *et al.*, 2013). In addition, the probability of normal full term pregnancy is reduced to 30%-50% in cancer survivors compared to the corresponding healthy general population (Chung *et al.*, 2013). 

Several options to restore ovarian function have been developed in the last years and are under assessment at present. These include preservation and transplantation of autologous ovarian fragments and/or whole ovaries into the patient, with no issues related to rejection or need for immunosuppression. However, since this procedure is largely devoted to cancer patients, there is the real danger that the ovarian tissue which is transplanted back may contains malignant cells, posing a severe risk to the patient (Meirow *et al.*, 2008; Dolmans *et al.*, 2010; Greve *et al.*, 2012).

**Figure 1:**
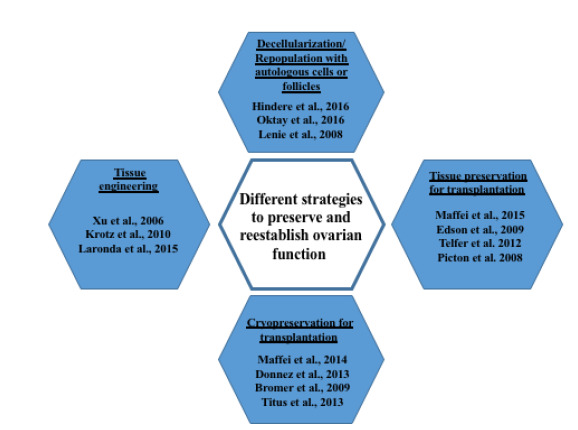
Different strategies presently used to restore ovarian function.

Tissue engineered ovarian scaffold and ovarian culture systems have been proposed as an alternative. This approach has been mainly designed to avoid the possibility to reintroduce the disease. It involves the use of 3D in vitro culture system, based on polymers of different sort and alginate and has allowed for the creation of artificial ovary scaffolds in vitro (Pangas *et al*, 2003; Xu *et al*, 2006). However most of the data available are limited to rodents. Furthermore, the biomaterials used alone lacked extracellular matrix (ECM) proteins that are essential to the phenotypes of mammalian cells (Huet *et al*., 2001; Che-Ying *et al*., 2016; Krotz *et al*., 2010). Because of this, ECM components such as arginine-glycine-aspartate (RGD), collagen (type I and IV), and/or fibronectin have been added to the matrix and led to improved growth, differentiation, and meiotic competence of the oocyte (Kreeger *et al*., 2003; Kreeger *et al*, 2006; Laronda *et al*., 2015). An experimental option is presently represented by the creation of biological scaffolds that are obtained through a decellularization process that removes all the original resident cells but leaves the ECM intact. This matrix is then repopulated with the patient cells, and can finally be transplanted. This protocol has been recently described and shown to successfully initiate puberty in mice (Laronda *et al*., 2015; Fig.1).

## Ovarian tissue preservation for transplantion strategies

Transplantation of cryopreserved ovarian follicles has been a standard clinical practice for a while, with relatively good success. This can be a vital strategy for young cancer patients who have to undergo chemotherapy and/or pelvic radiotherapy and typically requires the transplant of autologous ovaries to areas outside the irradiation area or maintained ex vivo until completion of chemotherapy. At remission, the preserved tissue can be returned to the donor. The treatment utilizes autologous ovaries so there are no issues related to rejection or need for immunosuppression. It can be greatly advantageous and indeed shown to allow recovery of fertility after total body irradiation and chemotherapy (Larsen *et al*., 2000; Rodriguez-Walleberg *et al*., 2015). However, preservation of functional activity during ex vivo maintenance has been a challenge tackled by the research of the last year, considering that the ovary is a complex organ that requires an intricate and dynamic three-dimensional structure in order to sustain follicle development and oocyte maturation (Picton *et al*., 2008; Edson *et al*., 2009; Telfer and McLaughlin, 2012). Numerous efforts have been dedicated to the design of culture protocols that allow optimal results. Nevertheless, with the exception of the mice, progress has been limited because of the longer period required for natural folliculogenesis, in larger species and for the related need for extended culture time to generate fully grown follicles and oocytes. A further problem affecting the functional ability of preserved tissue is the difficulty to mimic in vitro the complex relationship that takes place physiologically with the surrounding stroma and with the other ovarian structures. A possible option is the perfusion of the whole ovary, so that its entire architecture can be maintained intact and the supply of hormones and other regulatory factors through the blood circulation can be mimicked by a pump. This approach, which was first demonstrated by early experiments in the mouse, rat and rabbit, was more recently applied to larger species has been as a tool to test the viability and function of cryopreserved whole ovaries. We recently developed a perfusion system that allowed us to maintain functional whole sheep ovaries for up to 4 days. The data generated in those experiments demonstrated that the protocol was able to preserve functional activity of both fresh and cryopreserved ovaries, with identical hormonal secretion levels and comparable estradiol and progesterone production. These results were supported by morphological evidence that showed very good preservation of structural features in fresh and frozen tissue. Altogether, the evidence obtained supported the concept that an extended*ex* *vivo*culture of whole ovaries in larger species is possible even after cryopreservation and could lead to the retrieval of competent oocytes in the near future. Furthermore, this culture system could be applied to complement current protocols of cryopreservation of ovarian tissue for preserving fertility of human patients that survive cancer and provides an*in vitro*relevant model for physiological and toxicological studies.

## Cryopreservation of ovarian tissue: conventional and directional freezing

This approach can be achieved through the excision and banking of whole organs or of fragments of the cortical region. It is however interesting to note that, whereas very low success was reported following the cryopreservation of whole human ovaries, 24 live babies have been obtained after transplantation of frozen-thawed ovarian fragments (Donnez *et al.*, 2013). This clearly suggests that the use of ovarian fragments is the method of choice even if, at present, the average functional life span of these samples ranges from 2 to 5 years (Bromer and Patrizio, 2009; Donnez *et al.*, 2013). In theory, the cryopreservation of whole ovaries followed by the vascular anastomosis of the ovarian pedicle of the thawed organ should be able to guarantee a better follicular reserve and a longer life span of the transplant. However, the data available do not point in this direction. We recently demonstrated that this is largely due to the damage caused by the freezing procedures applied and showed that both ovarian structure and function were significantly better preserved when selecting an appropriate freezing procedure (Maffei *et al.*, 2014). In particular, we could show that directional freezing was able to significantly improved the integrity of all follicular structure, by maintaining a precise cooling rate and thus preventing the formation of intracellular ice. 

Directional freezing was beneficial for both entire ovaries and ovarian fragments and the results obtained clearly indicated a significant improvement versus the use of conventional cryopreservation strategies. We observed a higher cell proliferation rate and a better morphology. Furthermore, when we examined the expression of Heat Shock Proteins (HSPs), which are among the best characterized responses to thermal stress, we detected significantly stimulated, over baseline levels, only in the case of conventional freezing. Even more interestingly, we could observe that DNA damage was significantly lower in directional freezing samples and was always followed by a more intense DNA repair activity with the formation of a Rad51-ssDNA nucleoﬁlament, which is an essential step allowing full DNA double helix integrity re-establishment (Seeber *et al.*, 2013).This is a very important advantage of directional freezing and is particularly relevant for long-term function after thawing, since DNA double strand break repair efficiency has been identified as an important determinant of oocyte aging in women (Titus *et al.*, 2013). 

**Figure 2:**
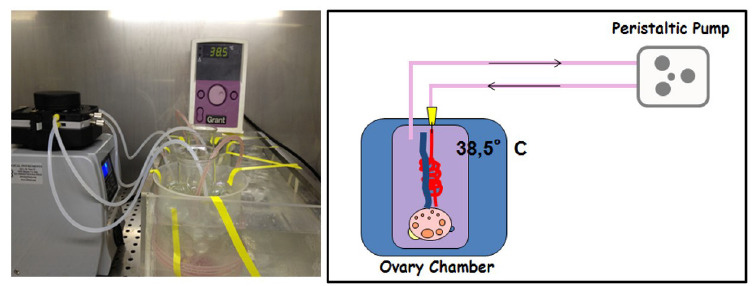
Perfusion systems allow to maintain functional whole sheep ovaries for several days, preserving functional activity of both fresh and cryopreserved organs, with identical hormonal secretion levels and comparable estradiol and progesterone production.

Altogether, we can affirm that directional freezing could represent a promising strategy for the preservation/transplantation of whole ovaries. However, the persistent technical difficulties linked to the surgical procedures required for re-transplantation is likely to still limit the use of whole organs in clinical settings. On the other hand the increasing concerns related to the danger of transferring malignant cells with transplanted frozen-thawed ovarian tissue may lead to a re-evaluation of the technique in terms of using whole ovaries as a source of high quality isolated follicles to be grown in artificial ovaries (Vanacker *et al.*, 2012; Fig. 2).

## Tissue engineering the ovary

Great efforts have been addressed lately in order to avoid reintroducing the disease in patients. In particular, scientists have developed tissue engineered oocyte maturation systems to activate oocytes isolated from cryopreserved ovarian tissues. Pangas*et al*. developed a 3D in vitro culture system with alginate beads that was used to grow individual granulosa cell-oocyte complexes (GOCs) and provided complexes with spatial arrangement (Xu *et al*., 2006). The alginate- based system was shown to yield far more oocytes than conventional IVF, as the majority of mammalian oocytes are contained in immature and quiescent follicles (Xu *et al*., 2006). Moreover, Xu *et al*. (2006) isolated immature follicles, cultured them in alginate droplet, fertilized in vitro and transferred to pseudo- pregnant female mice. They were able to generate viable offspring, of both female and male gender and fertile (Xu *et al*., 2006). 

Very promising results were also obtained using agarose, which is another polysaccharide-based biomaterial, widely used in tissue engineering, and that was used as a matrix for in vitro maturation of oocytes (Che-Ying *et al*., 2016). The three follicular cell types-theca, granulosa, and oocytes were homed in agarose to create a 3D artificial human ovary that was shown to allow successful maturation of early antral follicle (<10 mm) oocytes into metaphase II oocytes (Che-Ying *et al*., 2016). Although very encouraging, these approaches that used polysaccharide biomaterials alone, provided a suboptimal environment because they lacked ECM proteins that are essential to the phenotypes of mammalian cells (Huet *et al*., 2001). Because of this, other studies utilizing biomaterials added with ECM components such as RGD, collagen (type I and IV), and/or fibronectin, demonstrated improved growth, differentiation, and meiotic competence of the oocyte (Kreeger *et al*., 2003; Kreeger *et al*., 2006; Laronda *et al*., 2015). Further advantages were recently derived, adopting 3D bio-printing techniques that allows the seeding and the culture of different cell types recreating a layer-by-layer environment that builds a cyto/histological architecture similar to the original tissue (Irvine *et al*., 2015). The creation of the supports printed with compatible materials that can then be repopulated with the specific cell subpopulations, or the direct printing of a cells embedded matrix are the two possible options presently available.

**Figure 3:**
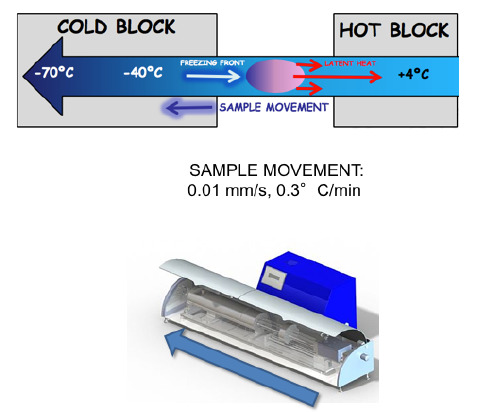
In directional freezing, the freezing rate is determined by the combination of temperature gradient and speed of the sample along the track. If the velocity of the sample is slower than the speed at which the heat is removed from the center of the sample towards its periphery, heat transfer is quickly removed in the direction opposite to that of the sample movement. All this results in a uniform cooling rate throughout the sample. The 3 thermal blocks are generally set at 4°, -10° and -70°C respectively, thereby imposing a temperature gradient around the tubes. Freezing tubes are pushed lengthwise, along the thermal gradient, and the speed is set at 0.01 mm/s, resulting in a cooling rate of 0.3°C/min down to -70°C. At the end of the procedure, samples are plunged into liquid nitrogen.

In the field of human and veterinary reproduction, the use of 3D printers is useful and necessary for the creation of ovarian prostheses or part of tissues supporting ovarian cells (Hinderer *et al*., 2016). In the near future bio-printing applied to reproductive medicine will be table to create a multi-layered functional culture, composed by different tissues and stratifications and develop a functional ovary with anatomical and physiological characteristic of the native organ (Shea *et al*., 2014). This may result in terrific advantages, restoring hormone and fertility function in onco-fertility patients. A very exciting report by Laronda *et al*. (2015) described the generation of a 3D-printed mouse model of ovarian follicles on a hydrogel support, with a compatible cyto-architecture like the original organ (Laronda *et al*., 2015). The follicle-seeded scaffolds became highly vascularized and was able to fully restore ovarian function when implanted in surgically sterilized mice. Moreover, pups were born through natural mating and thrive through maternal lactation. These findings indicate the possibility to create an*in vivo*functional ovarian implant, designed with 3D printing, and confirm tissue engineering as a tremendously promising strategy for regenerative reproductive biology (Fig. 3). 

## Decellularization/repopulation of ovary with autologous cells or follicles

**Figure 4:**
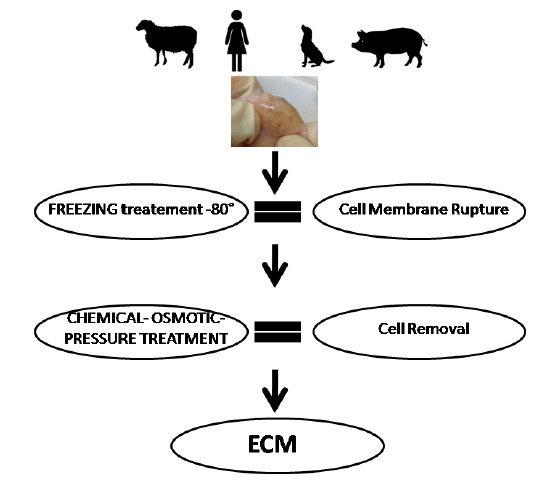
Schematic study plan to obtain a decellularized extracellular matrix from ovary. From the fresh ovary to the ECM material, the tissue is passed through the thermic treatment to obtain cell rupture. Effective cell removal is achieved thanks to the use of different ionic and non-ionic detergents, such as Triton X100, SDS and Deoxycolic acid and combining them to basic compounds such as ammonium hydroxide.

The field of regenerative medicine and tissue engineering has been developing for decades different techniques that promote and allow the proliferation of cells belonging to different tissues. One of these is the methodology of organ decellularization, a real removal of cells from parenchyma and non-parenchyma organs. The consequence of this method is to create a support and a scaffold for a subsequent homing of variegated cell types (Oktay *et al*., 2000; Shea *et al*., 2014; Hinderer *et al*., 2016). During the static or dynamic decellularization processes, the portion of tissue known as ECM is produced. There are several reports describing methodologies and applications to obtain a native and pure ECM without creating structural damage, thus obtaining an unusable scaffold. Remarkable capacity to promote cellular and tissues regeneration in various organs such as kidney, heart, lung and liver have been studied and described but few studies developed and showed this particular technique and its important role in primordial follicle growth and survival (Oktem and Oktay, 2007, Tapias and ott, 2014; Pati *et al*., 2014; Quian *et al*., 2015; Oktay *et al*., 2016). Indeed, the new field of ovary and uterus decellularization is developing continuously, filling the gap between the laboratory practice and the reproductive organ and cell needs in clinical transplantation. Moreover, the developed capacity to create anad hoc organ or 3D cell culture using the decellularization technique is evolving the field of Precise Medicine applications. For this reason the main scope of the decellularization is the ability to use the own patient cells and genetic kit to avoid and solve the obstacle of the organ rejection or data translation from in vitro to in vivo applications (Gilbert *et al*., 2006; Keane *et al*., 2015). The main point is to create ECM-based biomaterials, comprising non water-soluble structural proteins as collagens, laminins, elastin or proteoglycans (PGs), in association with important hormones and growth factors, used as stimulating compound, to better increase cell proliferation and organoid growth (Hinderer and Schenke-Layland K. 2013). To obtain the first native ECM material, different physical treatments such as agitation, sonication, mechanical pressure, or freeze-thawing procedures, let to disrupt cell membrane, in combination with washing solutions as deionized water and various ionic or non-ionic detergents (Andrée *et al*., 2014). Enzymatic treatment using trypsin, dispase, esterases or nucleases has also been employed to remove cells from tissues, becoming a part of the standard protocol used in daily lab. The ECM obtained with the different approaches described above, is utilized as a 3D fibrous and porous scaffold, with original nets and vasculature preserved. Needless to say, all the procedures finalized to remove the original cells from the organ has to avoid the deterioration of the matrix components (Hinderer *et al*., 2013). Most of the reports available, utilize ultrastructural analyses to evaluate the impact of the decellularization method chosen in damaging the crucial structural tissue elements such as elastic fiber (Lenie *et al*., 2008). For instance, PGs and glycosaminoglycans, which connect cells to collagen bundles or elastic fibers and induce a variety of cellular responses, must be fully preserved in decellularized tissues (Paulose *et al*., 2012), to allow for coordinated growth of the multiple cellular compartments of the follicle-the oocyte, granulosa cells, and theca cells-in vitro. The techniques used in our laboratory are selected based on the idea to cause the lesser mechanical and chemical stress for the biomatrix, but, at the same time, generate efficiently decellularized ECM. These involve the use of gradual temperature shock following a chemical treatment with Triton X and sodium dodecyl sulfate (SDS), utilizing a peristaltic pump with a low flow, low pressure and long perfusion time (Ghiringhelli *et al*., 2017). This experimental strategy allows for the obtainment of a well-preserved bio-scaffold that may constitute a promising environment to support the dynamic ovarian environment and the optimal niche for follicle maturation. The future prospective of ovary transplantation could be supported and implemented by all type of decellularized ECM materials, giving the chance to recreate new functional ovary and, avoiding the difficult obstacle of the short organ donors and the rejection during ovarian transplantation (Fig. 4). 

## Conclusions

The ovarian environment is complex and dynamic, it is modulated by cyclic changes in endocrine factors, both deriving from the systemic circulation as well as from local signals. In addition, the function and properties exerted by the ECM further contribute to the interactions with the follicle and its somatic cells14,15. Different bioengineering strategies have been presently adopted and shown to have great potential in order to preserve, maintain and or recreate the ovarian milieau. Transplantation of fresh or cryopreserved organs/fragments that re-establish ovarian function have been recently paired with the use of different and complex combination of cells, biomaterials and factors that stimulate local ability to regenerate the reproductive organ and methods that employ decellularization/repopulation of ovary with autologous cells or follicles. All these different options represent interesting, although still experimental alternatives to preserve or improve female fertility and are very promising strategies of regenerative reproductive medicine.
